# Supplementary figures and images for: HCGAN: hierarchical contrast generative adversarial network for unpaired sketch face synthesis
Source: PeerJ Comput Sci. 2024 Jul 31;10:e2184. doi: 10.7717/peerj-cs.2184 (PMC11322990; doi:10.7717/peerj-cs.2184)

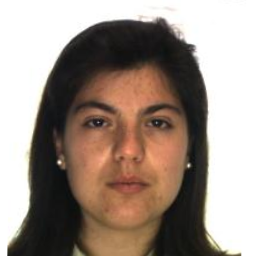

Supplement: Supplemental Information 2 — One of the dataset compression packages. [file peerj-cs-10-2184-s002.zip › AR/testA/img_080_aligned.png]

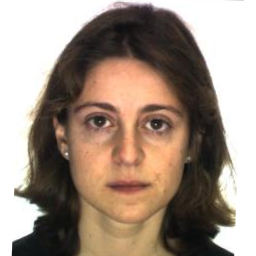

Supplement: Supplemental Information 2 — One of the dataset compression packages. [file peerj-cs-10-2184-s002.zip › AR/testA/img_081_aligned.png]

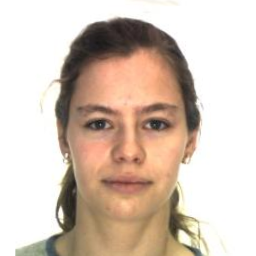

Supplement: Supplemental Information 2 — One of the dataset compression packages. [file peerj-cs-10-2184-s002.zip › AR/testA/img_082_aligned.png]

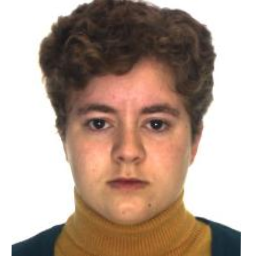

Supplement: Supplemental Information 2 — One of the dataset compression packages. [file peerj-cs-10-2184-s002.zip › AR/testA/img_083_aligned.png]

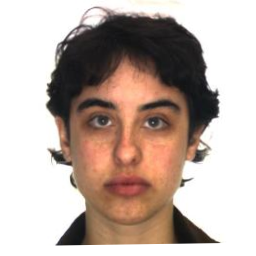

Supplement: Supplemental Information 2 — One of the dataset compression packages. [file peerj-cs-10-2184-s002.zip › AR/testA/img_084_aligned.png]

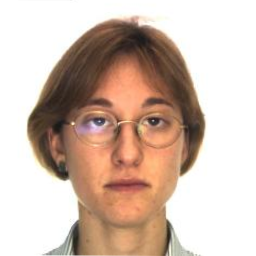

Supplement: Supplemental Information 2 — One of the dataset compression packages. [file peerj-cs-10-2184-s002.zip › AR/testA/img_085_aligned.png]

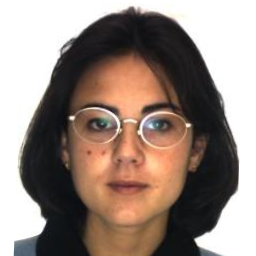

Supplement: Supplemental Information 2 — One of the dataset compression packages. [file peerj-cs-10-2184-s002.zip › AR/testA/img_086_aligned.png]

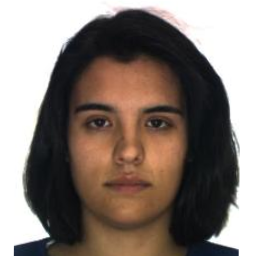

Supplement: Supplemental Information 2 — One of the dataset compression packages. [file peerj-cs-10-2184-s002.zip › AR/testA/img_087_aligned.png]

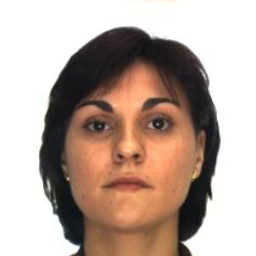

Supplement: Supplemental Information 2 — One of the dataset compression packages. [file peerj-cs-10-2184-s002.zip › AR/testA/img_088_aligned.png]

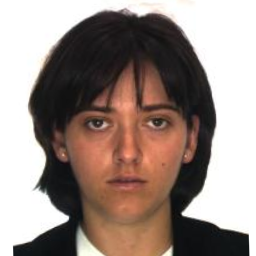

Supplement: Supplemental Information 2 — One of the dataset compression packages. [file peerj-cs-10-2184-s002.zip › AR/testA/img_089_aligned.png]

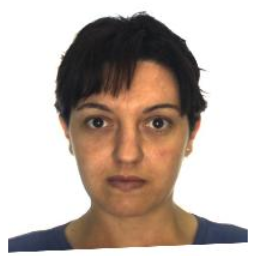

Supplement: Supplemental Information 2 — One of the dataset compression packages. [file peerj-cs-10-2184-s002.zip › AR/testA/img_090_aligned.png]

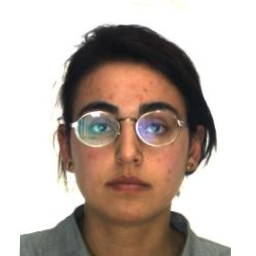

Supplement: Supplemental Information 2 — One of the dataset compression packages. [file peerj-cs-10-2184-s002.zip › AR/testA/img_091_aligned.png]

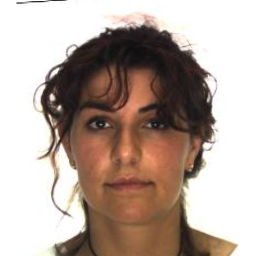

Supplement: Supplemental Information 2 — One of the dataset compression packages. [file peerj-cs-10-2184-s002.zip › AR/testA/img_092_aligned.png]

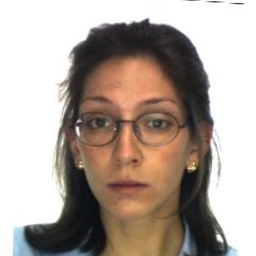

Supplement: Supplemental Information 2 — One of the dataset compression packages. [file peerj-cs-10-2184-s002.zip › AR/testA/img_093_aligned.png]

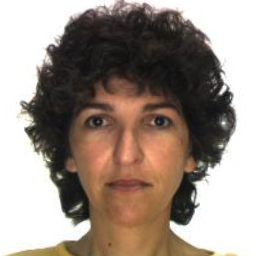

Supplement: Supplemental Information 2 — One of the dataset compression packages. [file peerj-cs-10-2184-s002.zip › AR/testA/img_094_aligned.png]

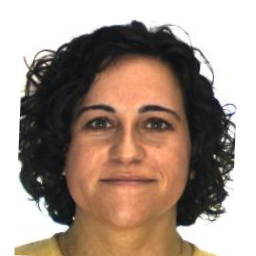

Supplement: Supplemental Information 2 — One of the dataset compression packages. [file peerj-cs-10-2184-s002.zip › AR/testA/img_095_aligned.png]

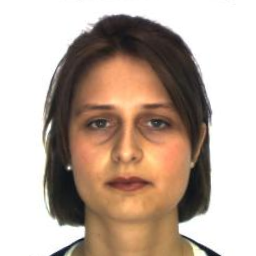

Supplement: Supplemental Information 2 — One of the dataset compression packages. [file peerj-cs-10-2184-s002.zip › AR/testA/img_096_aligned.png]

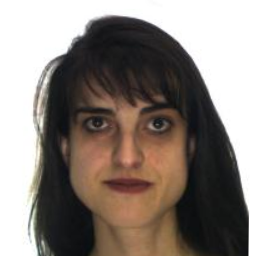

Supplement: Supplemental Information 2 — One of the dataset compression packages. [file peerj-cs-10-2184-s002.zip › AR/testA/img_097_aligned.png]

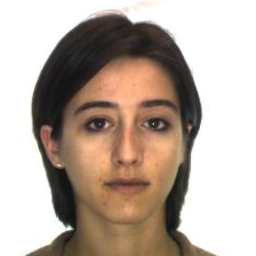

Supplement: Supplemental Information 2 — One of the dataset compression packages. [file peerj-cs-10-2184-s002.zip › AR/testA/img_098_aligned.png]

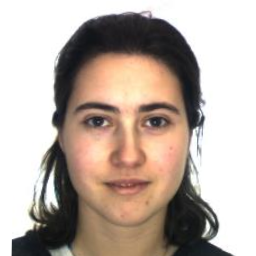

Supplement: Supplemental Information 2 — One of the dataset compression packages. [file peerj-cs-10-2184-s002.zip › AR/testA/img_099_aligned.png]

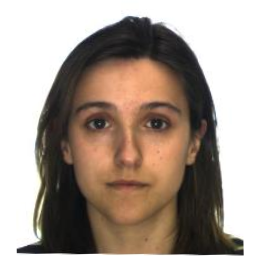

Supplement: Supplemental Information 2 — One of the dataset compression packages. [file peerj-cs-10-2184-s002.zip › AR/testA/img_100_aligned.png]

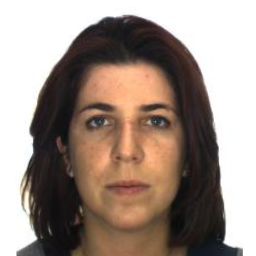

Supplement: Supplemental Information 2 — One of the dataset compression packages. [file peerj-cs-10-2184-s002.zip › AR/testA/img_101_aligned.png]

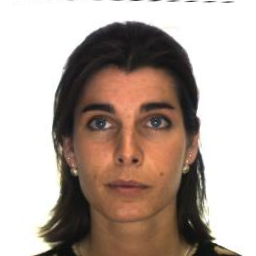

Supplement: Supplemental Information 2 — One of the dataset compression packages. [file peerj-cs-10-2184-s002.zip › AR/testA/img_102_aligned.png]

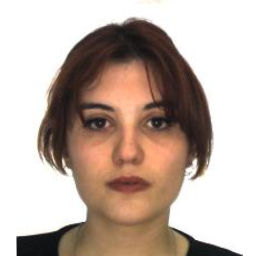

Supplement: Supplemental Information 2 — One of the dataset compression packages. [file peerj-cs-10-2184-s002.zip › AR/testA/img_103_aligned.png]

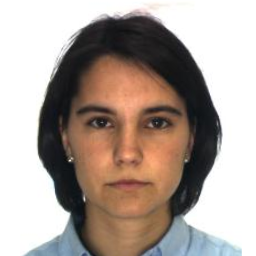

Supplement: Supplemental Information 2 — One of the dataset compression packages. [file peerj-cs-10-2184-s002.zip › AR/testA/img_104_aligned.png]

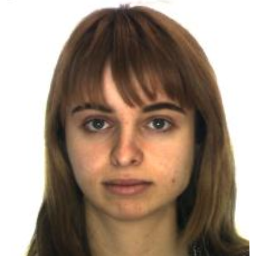

Supplement: Supplemental Information 2 — One of the dataset compression packages. [file peerj-cs-10-2184-s002.zip › AR/testA/img_105_aligned.png]

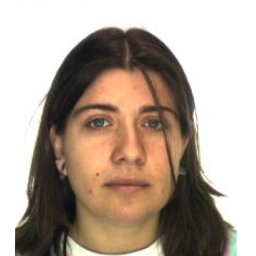

Supplement: Supplemental Information 2 — One of the dataset compression packages. [file peerj-cs-10-2184-s002.zip › AR/testA/img_106_aligned.png]

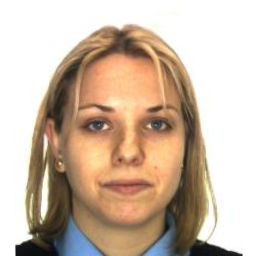

Supplement: Supplemental Information 2 — One of the dataset compression packages. [file peerj-cs-10-2184-s002.zip › AR/testA/img_107_aligned.png]

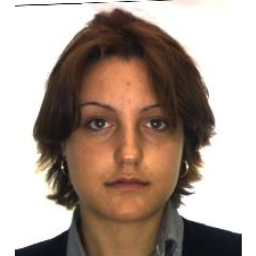

Supplement: Supplemental Information 2 — One of the dataset compression packages. [file peerj-cs-10-2184-s002.zip › AR/testA/img_108_aligned.png]

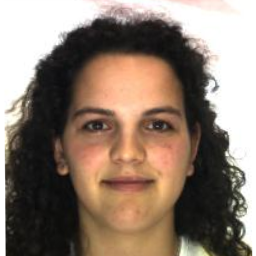

Supplement: Supplemental Information 2 — One of the dataset compression packages. [file peerj-cs-10-2184-s002.zip › AR/testA/img_109_aligned.png]

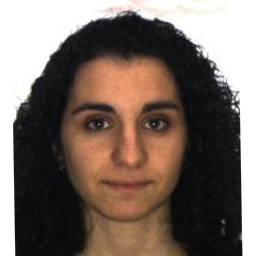

Supplement: Supplemental Information 2 — One of the dataset compression packages. [file peerj-cs-10-2184-s002.zip › AR/testA/img_110_aligned.png]

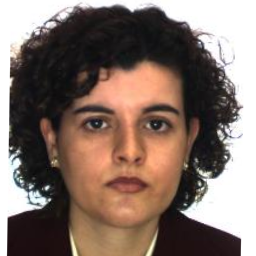

Supplement: Supplemental Information 2 — One of the dataset compression packages. [file peerj-cs-10-2184-s002.zip › AR/testA/img_111_aligned.png]

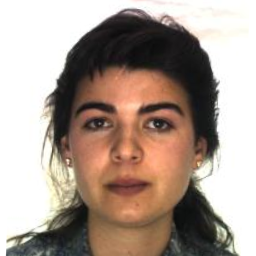

Supplement: Supplemental Information 2 — One of the dataset compression packages. [file peerj-cs-10-2184-s002.zip › AR/testA/img_112_aligned.png]

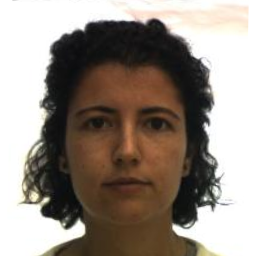

Supplement: Supplemental Information 2 — One of the dataset compression packages. [file peerj-cs-10-2184-s002.zip › AR/testA/img_113_aligned.png]

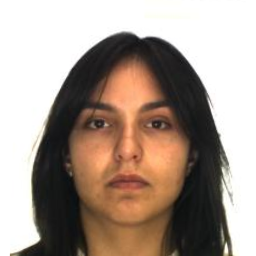

Supplement: Supplemental Information 2 — One of the dataset compression packages. [file peerj-cs-10-2184-s002.zip › AR/testA/img_114_aligned.png]

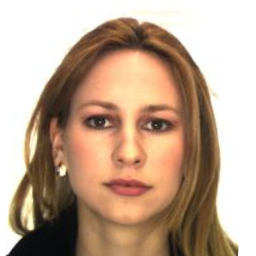

Supplement: Supplemental Information 2 — One of the dataset compression packages. [file peerj-cs-10-2184-s002.zip › AR/testA/img_115_aligned.png]

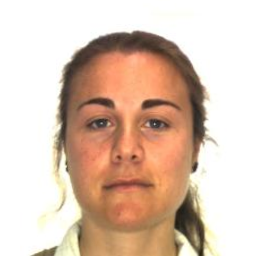

Supplement: Supplemental Information 2 — One of the dataset compression packages. [file peerj-cs-10-2184-s002.zip › AR/testA/img_116_aligned.png]

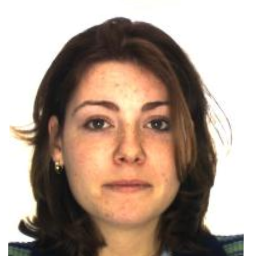

Supplement: Supplemental Information 2 — One of the dataset compression packages. [file peerj-cs-10-2184-s002.zip › AR/testA/img_117_aligned.png]

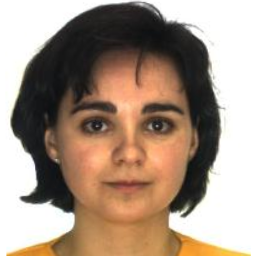

Supplement: Supplemental Information 2 — One of the dataset compression packages. [file peerj-cs-10-2184-s002.zip › AR/testA/img_118_aligned.png]

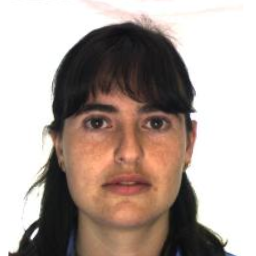

Supplement: Supplemental Information 2 — One of the dataset compression packages. [file peerj-cs-10-2184-s002.zip › AR/testA/img_119_aligned.png]

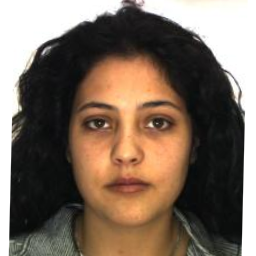

Supplement: Supplemental Information 2 — One of the dataset compression packages. [file peerj-cs-10-2184-s002.zip › AR/testA/img_120_aligned.png]

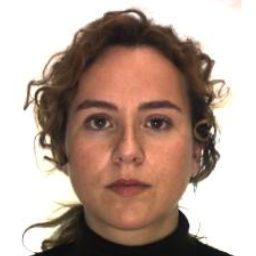

Supplement: Supplemental Information 2 — One of the dataset compression packages. [file peerj-cs-10-2184-s002.zip › AR/testA/img_121_aligned.png]

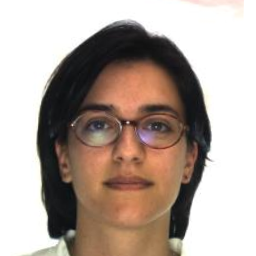

Supplement: Supplemental Information 2 — One of the dataset compression packages. [file peerj-cs-10-2184-s002.zip › AR/testA/img_122_aligned.png]

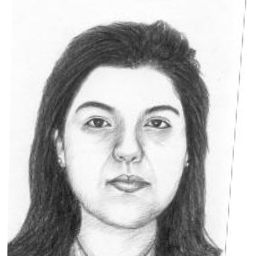

Supplement: Supplemental Information 2 — One of the dataset compression packages. [file peerj-cs-10-2184-s002.zip › AR/testB/img_080_aligned.png]

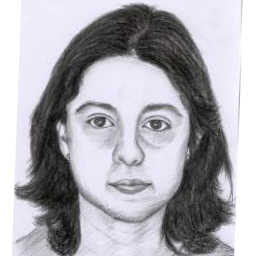

Supplement: Supplemental Information 2 — One of the dataset compression packages. [file peerj-cs-10-2184-s002.zip › AR/testB/img_081_aligned.png]

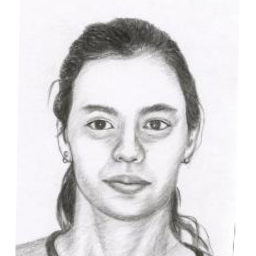

Supplement: Supplemental Information 2 — One of the dataset compression packages. [file peerj-cs-10-2184-s002.zip › AR/testB/img_082_aligned.png]

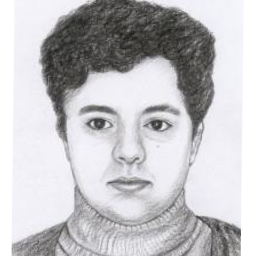

Supplement: Supplemental Information 2 — One of the dataset compression packages. [file peerj-cs-10-2184-s002.zip › AR/testB/img_083_aligned.png]

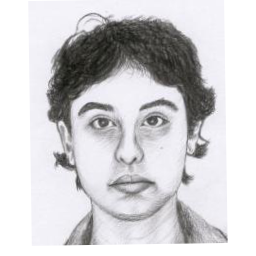

Supplement: Supplemental Information 2 — One of the dataset compression packages. [file peerj-cs-10-2184-s002.zip › AR/testB/img_084_aligned.png]

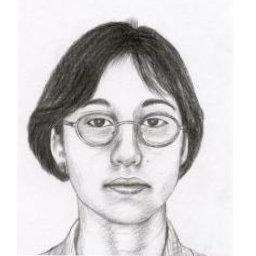

Supplement: Supplemental Information 2 — One of the dataset compression packages. [file peerj-cs-10-2184-s002.zip › AR/testB/img_085_aligned.png]

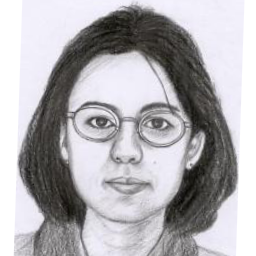

Supplement: Supplemental Information 2 — One of the dataset compression packages. [file peerj-cs-10-2184-s002.zip › AR/testB/img_086_aligned.png]

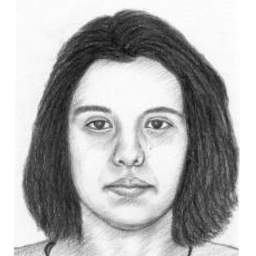

Supplement: Supplemental Information 2 — One of the dataset compression packages. [file peerj-cs-10-2184-s002.zip › AR/testB/img_087_aligned.png]

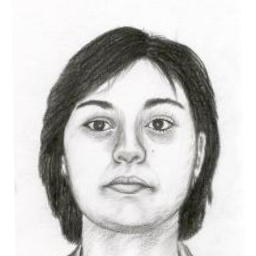

Supplement: Supplemental Information 2 — One of the dataset compression packages. [file peerj-cs-10-2184-s002.zip › AR/testB/img_088_aligned.png]

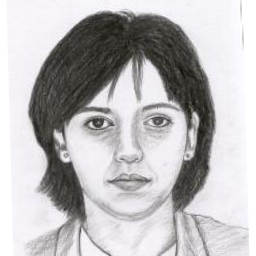

Supplement: Supplemental Information 2 — One of the dataset compression packages. [file peerj-cs-10-2184-s002.zip › AR/testB/img_089_aligned.png]

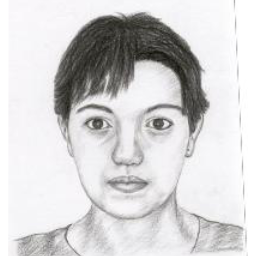

Supplement: Supplemental Information 2 — One of the dataset compression packages. [file peerj-cs-10-2184-s002.zip › AR/testB/img_090_aligned.png]

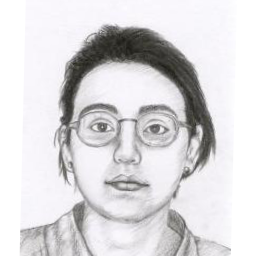

Supplement: Supplemental Information 2 — One of the dataset compression packages. [file peerj-cs-10-2184-s002.zip › AR/testB/img_091_aligned.png]

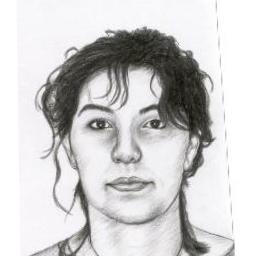

Supplement: Supplemental Information 2 — One of the dataset compression packages. [file peerj-cs-10-2184-s002.zip › AR/testB/img_092_aligned.png]

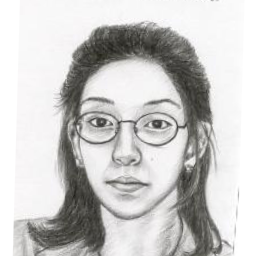

Supplement: Supplemental Information 2 — One of the dataset compression packages. [file peerj-cs-10-2184-s002.zip › AR/testB/img_093_aligned.png]

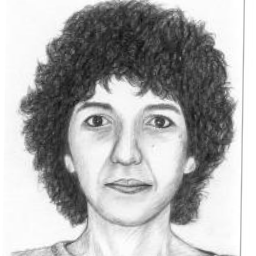

Supplement: Supplemental Information 2 — One of the dataset compression packages. [file peerj-cs-10-2184-s002.zip › AR/testB/img_094_aligned.png]

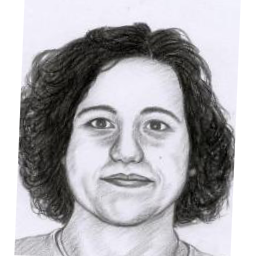

Supplement: Supplemental Information 2 — One of the dataset compression packages. [file peerj-cs-10-2184-s002.zip › AR/testB/img_095_aligned.png]

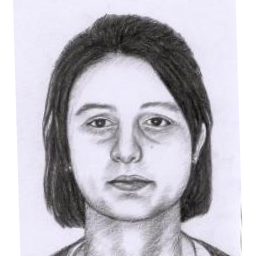

Supplement: Supplemental Information 2 — One of the dataset compression packages. [file peerj-cs-10-2184-s002.zip › AR/testB/img_096_aligned.png]

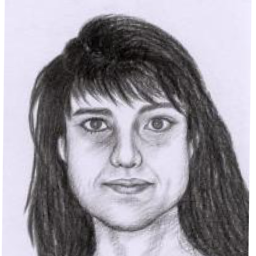

Supplement: Supplemental Information 2 — One of the dataset compression packages. [file peerj-cs-10-2184-s002.zip › AR/testB/img_097_aligned.png]

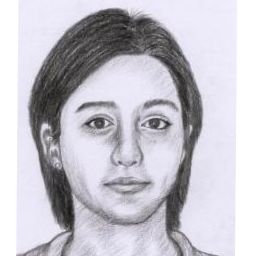

Supplement: Supplemental Information 2 — One of the dataset compression packages. [file peerj-cs-10-2184-s002.zip › AR/testB/img_098_aligned.png]

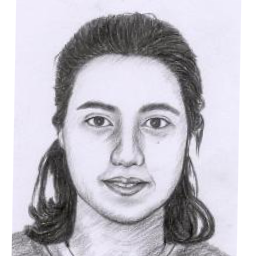

Supplement: Supplemental Information 2 — One of the dataset compression packages. [file peerj-cs-10-2184-s002.zip › AR/testB/img_099_aligned.png]

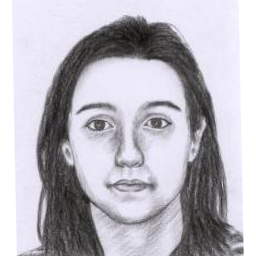

Supplement: Supplemental Information 2 — One of the dataset compression packages. [file peerj-cs-10-2184-s002.zip › AR/testB/img_100_aligned.png]

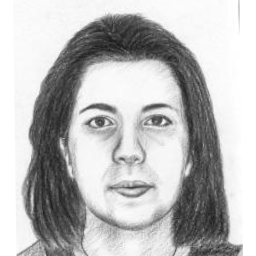

Supplement: Supplemental Information 2 — One of the dataset compression packages. [file peerj-cs-10-2184-s002.zip › AR/testB/img_101_aligned.png]

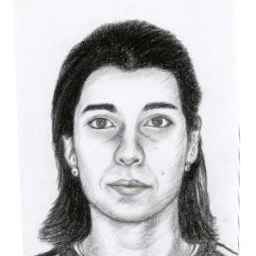

Supplement: Supplemental Information 2 — One of the dataset compression packages. [file peerj-cs-10-2184-s002.zip › AR/testB/img_102_aligned.png]

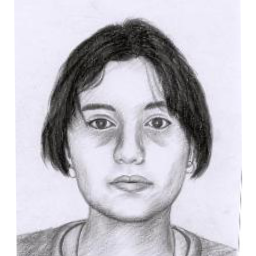

Supplement: Supplemental Information 2 — One of the dataset compression packages. [file peerj-cs-10-2184-s002.zip › AR/testB/img_103_aligned.png]

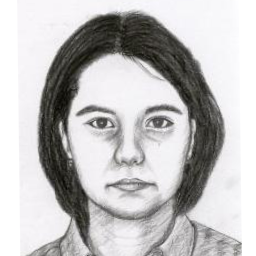

Supplement: Supplemental Information 2 — One of the dataset compression packages. [file peerj-cs-10-2184-s002.zip › AR/testB/img_104_aligned.png]

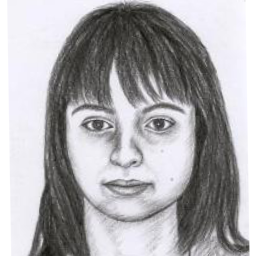

Supplement: Supplemental Information 2 — One of the dataset compression packages. [file peerj-cs-10-2184-s002.zip › AR/testB/img_105_aligned.png]

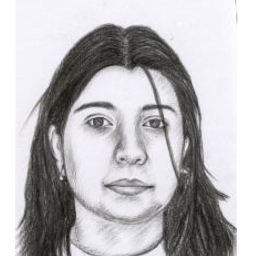

Supplement: Supplemental Information 2 — One of the dataset compression packages. [file peerj-cs-10-2184-s002.zip › AR/testB/img_106_aligned.png]

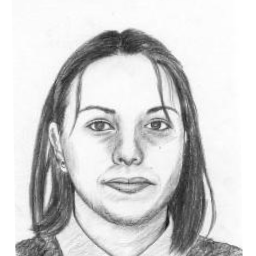

Supplement: Supplemental Information 2 — One of the dataset compression packages. [file peerj-cs-10-2184-s002.zip › AR/testB/img_107_aligned.png]

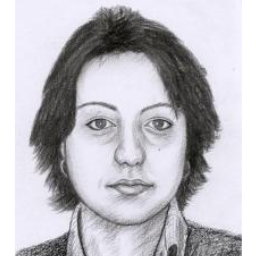

Supplement: Supplemental Information 2 — One of the dataset compression packages. [file peerj-cs-10-2184-s002.zip › AR/testB/img_108_aligned.png]

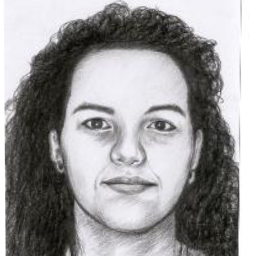

Supplement: Supplemental Information 2 — One of the dataset compression packages. [file peerj-cs-10-2184-s002.zip › AR/testB/img_109_aligned.png]

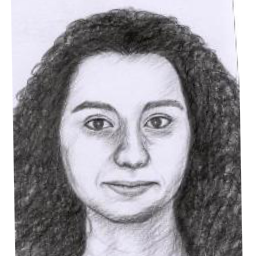

Supplement: Supplemental Information 2 — One of the dataset compression packages. [file peerj-cs-10-2184-s002.zip › AR/testB/img_110_aligned.png]

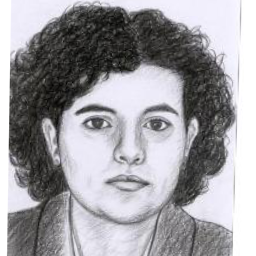

Supplement: Supplemental Information 2 — One of the dataset compression packages. [file peerj-cs-10-2184-s002.zip › AR/testB/img_111_aligned.png]

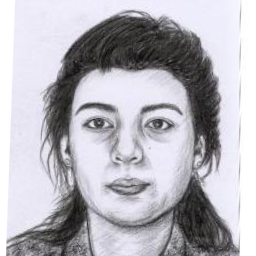

Supplement: Supplemental Information 2 — One of the dataset compression packages. [file peerj-cs-10-2184-s002.zip › AR/testB/img_112_aligned.png]

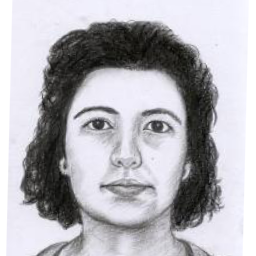

Supplement: Supplemental Information 2 — One of the dataset compression packages. [file peerj-cs-10-2184-s002.zip › AR/testB/img_113_aligned.png]

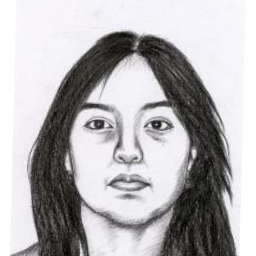

Supplement: Supplemental Information 2 — One of the dataset compression packages. [file peerj-cs-10-2184-s002.zip › AR/testB/img_114_aligned.png]

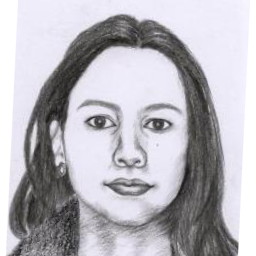

Supplement: Supplemental Information 2 — One of the dataset compression packages. [file peerj-cs-10-2184-s002.zip › AR/testB/img_115_aligned.png]

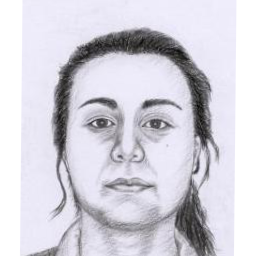

Supplement: Supplemental Information 2 — One of the dataset compression packages. [file peerj-cs-10-2184-s002.zip › AR/testB/img_116_aligned.png]

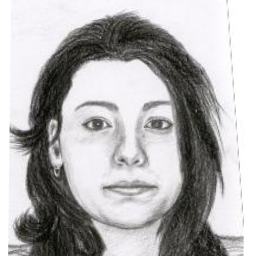

Supplement: Supplemental Information 2 — One of the dataset compression packages. [file peerj-cs-10-2184-s002.zip › AR/testB/img_117_aligned.png]

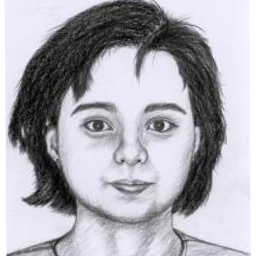

Supplement: Supplemental Information 2 — One of the dataset compression packages. [file peerj-cs-10-2184-s002.zip › AR/testB/img_118_aligned.png]

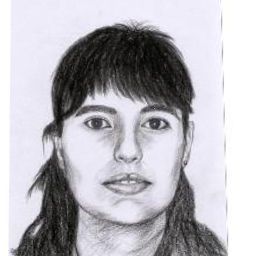

Supplement: Supplemental Information 2 — One of the dataset compression packages. [file peerj-cs-10-2184-s002.zip › AR/testB/img_119_aligned.png]

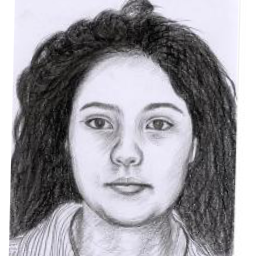

Supplement: Supplemental Information 2 — One of the dataset compression packages. [file peerj-cs-10-2184-s002.zip › AR/testB/img_120_aligned.png]

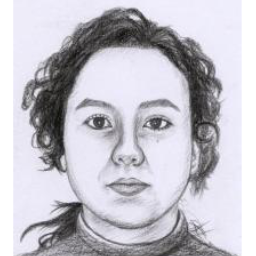

Supplement: Supplemental Information 2 — One of the dataset compression packages. [file peerj-cs-10-2184-s002.zip › AR/testB/img_121_aligned.png]

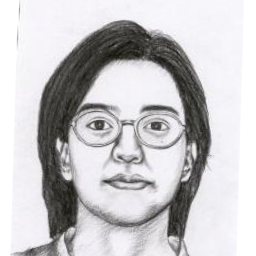

Supplement: Supplemental Information 2 — One of the dataset compression packages. [file peerj-cs-10-2184-s002.zip › AR/testB/img_122_aligned.png]
